# Structural validity and internal consistency of Picture My Participation: A measure for children with disability

**DOI:** 10.4102/ajod.v10i0.763

**Published:** 2021-05-28

**Authors:** Patrik Arvidsson, Shakila Dada, Mats Granlund, Christine Imms, Lin Jun Shi, Lin Ju Kang, Ai-Wen Hwang, Karina Huus

**Affiliations:** 1 Children, Health, Intervention, Learning and Development (CHILD), Faculty of Health Science, Jönköping University, Jönköping, Sweden; 2 Swedish Institute for Disability Research, School of Health and Welfare, Faculty of Health and Welfare, Jönköping University, Jönköping, Sweden; 3 Centre for Research & Development, Uppsala University, Gävleborg, Sweden; 4 Centre for Augmentative and Alternative Communication, Faculty of Humanities, University of Pretoria, Pretoria, South Africa; 5 Department of Paediatrics, Faculty of Medical, Dental and Health Sciences, The University of Melbourne, Melbourne, Australia; 6 School of Nursing, Tianjin Medical University, Heping District, China; 7 Graduate Institute of Early Intervention, Chang Gung University, Tao-Yuan, Taiwan; 8 The Department of Physical Medicine and Rehabilitation, Chang Gung Memorial Hospital, Linkou, Taiwan

**Keywords:** low- and middle-income countries, everyday functioning, picture supported Interview, cognitive support, self-ratings

## Abstract

**Background:**

Picture My Participation (PMP) intended to measure participation, defined as attendance and involvement in everyday situations, of children with disabilities, particularly in low- and middle-income settings.

**Objectives:**

To explore structural validity of PMP by identifying possible subcomponents in the attendance scale and examining internal consistency of the total score and each subcomponent.

**Method:**

A picture-supported interview was conducted with 182 children, 7–18 years, with and without intellectual disability (ID). Frequency of attendance in 20 activities was rated on a four-point Likert scale (never, seldom, sometimes and always).

**Results:**

An exploratory principal component analysis extracted four subcomponents: (1) organised activities, (2) social activities and taking care of others, (3) family life activities and 4) personal care and development activities. Internal consistency for the total scale (alpha = 0.85) and the first two subcomponents (alpha = 0.72 and 0.75) was acceptable. The two last subcomponents alpha values were 0.57 and 0.49.

**Conclusion:**

The four possible subcomponents of PMP can be used to provide information about possible domains in which participation and participation restrictions exist. This study provided further psychometric evidence about PMP as a measure of participation. The stability and the utility of these subcomponents needed further exploration.

## Introduction

The participation construct is considered as an essential reflector of an individual actual function in real life and should therefore be researched in clinical practices and specifically in relation to disability and health (United Nations 2006; United Nations General Assembly 1989; World Health Organization [WHO] 2001, 2007). Participation and participation restrictions, defined as involvement and problems with involvement in everyday activities, respectively, are key components of social inclusion and exclusion (United Nations 2006; United Nations General Assembly 1989; WHO 2001, 2007). Whilst participation is generally agreed to be an important outcome for children, there is a lack of appropriate self-report measures of participation for children (Adair et al. 2018; Rainey et al. 2014). Article 12 of the Convention of Children’s rights (United Nations General Assembly 1989) highlights the right of every child to formulate their own opinions and express them freely in accordance with their maturity and age. However, whenever information on children was included in research concerning low- and middle-income countries (LMICs), researchers have relied mainly on proxy ratings by adults in these children’s lives (Carroll-Lind, Chapman & Raskauskas 2011; Lygnegard et al. 2013; Schlebusch et al. 2020). Exclusion of the opinions of children with complex disabilities, including cognitive impairment and communication difficulties associated with autism and intellectual disability (ID) (American Psychiatric Association [APA] 2013), is particularly evident, because they are often dependent on proxy persons to express themselves. A child’s rights perspective demands children to be asked about their own experiences and perceptions, even if they have an intellectual impairment or communication difficulties (Huus et al.2015; Oosterhoom & Kendrick 2001).

When endorsing an integrative and multidimensional understanding of functioning and health for individuals with disability, participation can be considered as a reflector of the interaction between body impairments and societal barriers (Arvidsson et al. 2014; Imms et al. 2017; WHO 2001, 2007). An integrative approach towards disability attempts to highlight actual interaction within their everyday context and consider support structures and any impairment (e.g. cognitive impairment) (American Association on Intellectual and Developmental Disabilities [AAIDD] 2010; Buntinx & Schalock 2010). For individuals with ID, the experience of participation can be assessed by self-ratings in everyday activities and can be operationalised by the frequency of attendance and/or intensity of involvement (Arvidsson et al. 2014; Arvidsson & Granlund 2018; Granlund et al. 2012; Huus et al. 2015). The attendance aspect is related to being able to be present in life situations or activity settings and is related to the individual’s right to be socially included and to actually take part in the same activities as any other citizen. The attendance aspect is also relevant when, for instance, focusing on participation at a group and/or societal level (Arvidsson et al. 2014; Granlund et al. 2012). The intensity of involvement is a reflector of how participation is actually experienced in the activity. Involvement is relevant when focusing on the experience of participation and thus for individual interventions, for example, in individuals with ID (Arvidsson et al. 2014; Arvidsson & Granlund 2018; Granlund et al. 2012; Huus et al. 2015).

Picture My Participation (PMP) is a self-report instrument that was specifically designed to capture the two aspects of participation, namely, attendance and perceived involvement in children and youth with mild ID in 20 different activities related to home, social life and community (Arvidsson et al. 2020; Bolton et al. 2020). The items were selected by reviewing existing participation measures, Participation and Environment Measure for Children and Youth (PEM-CY), Children’s Assessment of Participation and Enjoyment and Preferences for Activities of Kids (CAPE) and matching items to the UN Convention of Children’s Rights (Khetani et al. 2014; King et al. 2004; Mandich et al. 2004). In addition, the selected items were reviewed in relation to resource-poor environments to identify areas that are not covered by measures, which are developed in high-income settings. Finally, items were linked to International Classification of Functioning, Disability and Health – Version for Children & Youth (ICF-CY) codes to make sure that the activities selected included activities that could be considered important and relevant in LMIC settings and representative of the activity and participation chapters of the ICF-CY (WHO 2007). The contents of the 20 activity items of the PMP instrument were found to be valid for children and youth living in an LMIC (in this case, South Africa), as well as for children and youth with ID in both an LMIC (South Africa) and a high-income country (HIC) (Sweden) (Arvidsson et al. 2020). Whilst the instrument seemed promising in terms of appropriate content, additional psychometric properties such as test-retest reliability, structural validity and internal consistency require exploration. The current paper focuses on aspects of structural validity of the PMP, as defined by Mokkink et al. (2010b:9), that is, the degree to which the scores of an instrument are an adequate reflection of the dimensionality of the construct to be measured. For the PMP, it applies to using the instrument as a tool for gathering knowledge about the attendance aspect of participation in different settings and in different countries. Participation is best conceptualised as the frequency of attendance in different activities and is best seen as a participation profile where attendance will vary between different types of activities or subcomponents (Arvidsson et al. 2020; Imms et al. 2017). Several studies, for example, Ullenhag et al. (2014) reported that children with disabilities tend to participate less in out of home activities than other children and also less in informal activities with peers. Thus, the main aim of this study was to explore the attendance aspect of PMP regarding structural validity by identifying and describing possible subcomponents, that is, type of activities for which indices could be created to obtain a participation profile. An additional aim was to explore the internal consistency for the attendance aspect of PMP (all 20 items of the attendance scale) and for the subcomponents (provided that subcomponents are identified by the structural validation). Participation instruments especially designed to fit for activities in low- and middle-income settings are lacking (Schlebusch et al. 2020). Evidence from this study is important for supporting decisions regarding whether, and how, scores from PMP can be collated to summarise participation attendance levels as a profile of type of activity usually seen in low-income settings.

## Materials and methods

### Design

This cross-sectional, instrument validation study was designed according to the COnsensus-based Standards for the selection of health Measurement INstruments (COSMIN) principles (Mokkink et al. 2010b) to explore the structural validity of the PMP and the internal consistency of the attendance scores.

### Settings

To obtain data on the utility of the PMP measure for countries with different cultures and income levels, data were collected in South Africa, Taiwan, Mainland China and Sweden. In South Africa, the study was conducted in a city of approximately 200 000 inhabitants, in Taiwan in one city with approximately 2.6 million inhabitants, in Mainland China in two cities with 7.6 and 15.6 million inhabitants, respectively, and in Sweden in two cities with approximately 100 000 inhabitants each.

### Participants

An instrument with universal utility is valid and reliable under different circumstances. The purpose of the sampling strategy was to ensure variation in the samples in terms of age, gender, country/context, socio-economic circumstances and level of disability. In addition to targeting samples from four different countries, we sought children with mild ID from all four countries. Children with typical development (TD) were for reasons of convenience recruited only in South Africa. Children with TD were recruited to obtain variation of participation also within low- and middle-income setting. Consequently, five subgroups of children were recruited: (1) children with ID in South Africa (*n* = 99), (2) children with TD in South Africa (*n* = 37), (3) children with ID in Mainland China (*n* = 20), (4) children with ID in Taiwan (*n* = 30) and (5) children with ID Sweden (*n* = 20). A total of 182 participants were recruited. Descriptive data for each subgroup and for all participants as a total are presented in Table 1.

**TABLE 1:** Descriptive data regarding gender, age and Picture My Participation total scores for the five subsamples and for all participants together.

Variables		Five subsamples					All participants together (*n* = 182)
		Children from South Africa with ID (*n* = 79)	Children from South Africa with TD (*n* = 33)	Children from Mainland China with ID (*n* = 20)	Children from Taiwan with ID (*n* = 30)	Children from Sweden with ID (*n* = 20)	
Gender, *n* (%)	Girls	36 (46%)	22 (67%)	8 (40.0 %)	11 (36.7 %)	6 (30.0 %)	83 (45.6 %)
	Boys	38 (48%)	11(33%)	12 (60.0%)	19 (63.3 %)	14 (70.0 %)	94 (51.6 %)
	Missing	5 (6%)	0	0	0	0	5 (2.7 %)
Age (years)	Min – Max	9 – 16	9 – 14	7 – 18	8 – 12	7 – 18	7 – 18
	Mean (s.d.)	12.7 (1.7)	11.2 (1.6)	12.3 (3.1)	10.5 (1.3)	11.7 (3.1)	11.9 (2.2)
	Missing (single items)	5	0	0	0	0	5 (2.7 %)
PMP total score (19 items) (Likert scale: 1–4 Score range: 19–76)	Min – Max	28 – 74	48 – 69	30 – 66	49 – 63	28 – 69	28 – 47
	Mean (s.d.)	53.2 (11.4)	60.8 (5.7)	47.5 (7.5)	49.4 (5.7)	51.3 (9.5)	52.4 (8.0)
	Missing (single items)	2	1	0	0	7	10

PMP, Picture My Participation; ID, intellectual disability; TD, typical development.

#### Inclusion criteria

Children were eligible for inclusion if they had been diagnosed with ID and attended a school for children with ID, as confirmed by their caregivers. Children with either ID or TD also needed to meet the following criteria to be included: (1) aged between 7 and 18 years, (2) able to speak and understand English (in South Africa), Swedish (in Sweden) or Mandarin (in Mainland China and Taiwan), and (3) assented to participate in the study. For all children, the legal caregiver had to give consent for their child to participate.

### Variables and measurements

All the data were collected by clinical researchers who conducted structured interviews or by specially trained postgraduate students with knowledge about the target group and the PMP. Data related to participant characteristics, including date of birth and gender, were collected using a parent-report survey.

#### Picture my Participation

The participation instrument PMP (Arvidsson & Granlund 2018) was back-translated (Ullenhag et al. 2014) from English into Swedish and Mandarin. This instrument is designed for children and youth aged from 5 to 21 years and it measures participation in 20 home and community activities (see Table 2). Furthermore, the PMP, which is administered as a picture-supported one-on-one interview with a child, comprises four sections:

1. frequency of attendance for each item, rated on a four-point Likert scale (never, seldom, sometimes and always)
2. selection of the three most important activities according to the child
3. perceived involvement (by the child) in these three activities, rated on a three-point Likert scale (not involved, somewhat involved and very involved). In this section, the children were also asked if there was any other activity that they would select as important, besides the 20 activities that were asked about in the PMP
4. evaluation of perceived barriers to and facilitators of participation in relation to the activities that were the most important to the children.

**TABLE 2:** Descriptive statistics of the principal component analysis based on all Picture My Participation items.

Variables	Frequencies of ratings					Mean score	s.d.
	Always (4)	Sometimes (3)	Seldom (2)	Never (1)	Missing N		
Personal care	136	32	4	4	0	3.7	0.63
Family mealtime	106	54	8	8	0	3.5	0.79
My own health	47	44	48	37	0	2.6	1.10
Gathering supplies	35	57	31	52	1	2.4	1.11
Meal preparation	29	48	38	59	2	2.3	1.10
Cleaning at home	45	73	33	24	1	2.8	0.98
Caring for family	56	52	27	39	2	2.7	1.13
Caring for animals/pets	46	22	30	78	0	2.2	1.26
Family time	90	49	27	10	0	3.2	0.92
Celebrations	57	63	39	17	0	2.9	0.96
Playing with others	50	63	30	33	0	2.7	1.07
Organised leisure	65	46	34	31	0	2.8	1.12
Quiet leisure	78	44	33	21	0	3.0	1.06
Spiritual activities	57	39	40	40	0	2.6	1.16
Shopping	46	67	35	27	1	2.8	1.01
Social activities	13	44	38	78	3	2.0	1.00
Health centre	28	72	57	19	0	2.6	0.88
School	131	33	4	8	0	3.6	0.74
Overnights visits and trips	52	68	39	17	0	2.9	0.95

s.d., standard deviation.

Administration took 20 to 30 min for each child. For the purposes of this study, only data from section 1 (frequency of attendance) were used.

### Data collection

The PMP was completed as part of structured interviews in which graphic symbols from the aided symbol set of Picture Communication Symbols (PCS™) were used (Fuller & Lloyd 1997). These symbols are available as part of the Boardmaker™ software program developed by Mayer-Johnson, LLC (Mayer-Johnson 2015). Picture Communication Symbols ™ were used during the child assent procedure and as part of the PMP instrument. A specific picture-supported interview approach, called Talking Mats™, was used (Cameron & Murphy 2002). The Talking Mats™ framework is a strategy to facilitate conversations with persons with disabilities and communication with children with ID. The strategy involves placing a mat (a piece of carpet measuring 49 cm × 34.5 cm) in front of the child. In the bottom section of the mat, the child can place their PCS™ symbols for the different activities to indicate their responses. Three trial items were provided to facilitate the children’s understanding of the attendance ratings and to ensure that they understood the instructions. The children were asked (with respect to each attendance construct), ‘How often do you participate in daily routines?’ and at the same time, they were shown the PCS™ symbol of the routines. For the ratings of attendance, one mat was divided into four equal columns using masking tape. The upper section contained the visual scale that represented the four-point Likert scale items, depicted with pictures of baskets of apples: ‘Never’ (showing an empty basket with no apples), ‘Seldom’ (showing a basket with two apples), ‘Sometimes’ (showing a basket with five apples) and ‘Always’ (showing a basket completely filled with apples). The child had to place the PCS™ symbol on the mat in the column to indicate the item that they felt best represented the frequency of their participation. The researcher recorded the child’s response on a separate score sheet and moved on to the next question until all 20 items were completed. Non-contingent feedback was provided. Data were collected in the same way for all five subgroups of participants.

### Data analysis

Participant characteristics were summarised descriptively. The four-point Likert scale for measuring attendance was prepared with the following values: 1 = never, 2 = seldom; 3 = sometimes, 4 = always; total scores were calculated by averaging responses to each item. Total scores were summarised descriptively for each subsample and for the total sample. All statistical analyses were performed using SPSS 24.0.

#### Principal component analysis

An exploratory principal component analysis (PCA) was used as the extraction method to explore the dimensionality of the scale and investigate possible subcomponents of the PMP. In this PCA, 19 of the 20 items were used. The item ‘paid and unpaid employment’ was excluded based on the experience of the data collectors. No child was attending any activity that could be considered as employment and most of the children were confused by the question. The rotation method used was Varimax with Kaiser Normalisation and the result was that the rotation converged in nine iterations, eigenvalues >1.

#### Subcomponents

Children’s attendance in different activities tends to vary between type of activities with certain types being more commonly attended universally (e.g. within family activities), whilst others may vary depending on economical circumstances (e.g. organised leisure activities outside home) (Arvidsson et al. 2020). The 19 PMP items comprising the identified four subcomponents were discussed to consider the theoretical and practical relevance of the item clustering and interpretability of the statistical result by a panel of 12 researchers. This multidisciplinary panel included the authors of this article and other researchers in the field of disability research and early childhood intervention. The similarities of the items within each of the four subcomponents and the differences between the four subcomponents were discussed. After a final discussion, the contents of the four subcomponents were described.

#### Internal consistency

Cronbach’s alpha coefficients were used to calculate the internal consistency of the total scale of the PMP and for any identified subcomponents. Alpha values greater than 0.70 are considered to demonstrate adequate internal consistency (Terwee et al. 2007).

### Ethical considerations

Ethical approval for the study was obtained from the Ethics Committees and Boards in each of the four participating countries (University of Pretoria , South Africa, reference number: GW20180301HS; Tianjin Medical University , People Republic of China, reference number: TUMEC20140201; Regionala etikprovningsnamnden, Sweden, reference number: 2017/234-32; Chang Gung Medical Foundation, IRB number: 201600861B0) and from the relevant local Departments of Education and school principals. Informed consent was obtained from every child’s primary caregiver and consent was also sought from every participating child in each of the countries involved in the study.

## Results

Participant characteristics are presented in Table 1. Descriptive statistics regarding PMP are based on total scores.

### Principal component analysis

Table 2 presents the descriptive statistics for each item. The steps followed in the exploratory PCA are shown in Tables 3–4. The Kaiser-Meyer-Olkin Measure of Sampling Adequacy was 0.841 and Bartlett’s Test of Sphericity showed approximate Chi-Square 729 425 (degrees of freedom [*df*]** 171, sig. < 0.001).

**FIGURE 1:**
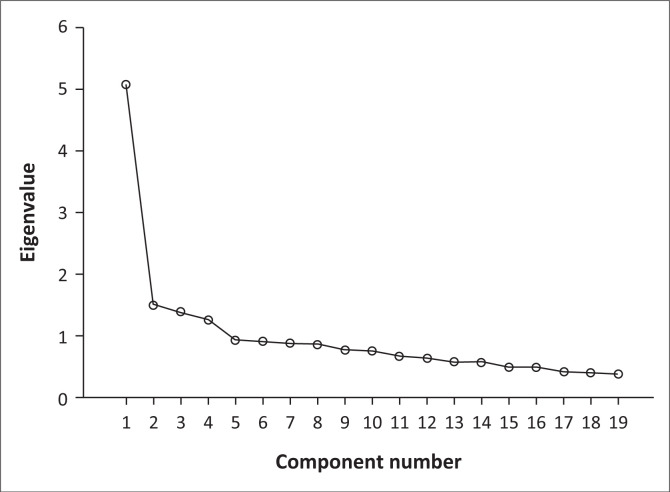
Component matrix demonstrating four components that are evident in the data set. Scree plot.

Principal component analysis extractions with total variance explained, with initial eigenvalues and after rotation, are presented in Table 3 and Figure 1. The PCA extracted four components from the children’s responses and these results are presented in Table 4. The four suggested subcomponents were labelled and described as follows.

**TABLE 3:** Principal component analysis extraction with total variance explained, with initial eigenvalues and after rotation.

PMP item	Initial Eigenvalues			Rotation sums of squared loadings		
	Total	% of variance	Cumulative %	Total	% of variance	Cumulative %
1	5.1	26.7	26.7	2.9	15.1	15.1
2	1.5	7.9	34.6	2.8	14.7	29.7
3	1.4	7.3	41.8	1.9	9.9	39.6
4	1.3	6.7	48.5	1.7	8.9	48.5
5	0.9	5.0	53.5	-	-	-
6	0.9	4.8	58.2	-	-	-
7	0.9	4.7	62.9	-	-	-
8	0.9	4.6	67.5	-	-	-
9	0.8	4.1	71.5	-	-	-
10	0.8	4.0	75.5	-	-	-
11	0.7	3.5	79.0	-	-	-
12	0.6	3.4	82.4	-	-	-
13	0.6	3.0	85.5	-	-	-
14	0.6	3.0	88.5	-	-	-
15	0.5	2.6	91.1	-	-	-
16	0.5	2.6	93.7	-	-	-
17	0.4	2.2	95.9	-	-	-
18	0.4	2.1	98.0	-	-	-
19	0.4	2.0	100.0	-	-	-

PMP, Picture My Participation.

**TABLE 4:** Principal component analysis – Rotated component matrix.

Variables	Component			
	1	2	3	4
Overnight visits and trips	0.702*	-	-	-
Organised leisure	0.654*	-	0.203	-
Cleaning at home	0.635*	0.120	0.143	0.205
Health centre	0.594*	0.348	-0.126	-
Gathering supplies	0.585*	0.120	0.185	0.106
Shopping	0.456*	0.312	0.173	0.154
Playing with others	-	0.678*	0.113	0.131
Caring for family	0.129	0.635*	-	0.300
Spiritual activities	0.138	0.621*	0.241	-
Celebrations	0.210	0.571*	-	0.257
Caring for animals/pets	0.413	0.523*	-0.267	-
Social activities	0.181	0.490*	0.344	-
Meal preparation	0.144	0.490*	0.436	-
Family time	-	0.355	0.685*	-
Family mealtime	0.175	-	0.649*	0.396
Quiet leisure	0.402	-	0.599*	
School	-	-	0.152	0.741*
Personal care	0.103	0.108	-	0.721*
My own health	0.355	0.236	-	0.414*

* , statistically significant.

#### Subcomponent 1: Organised activities

This subcomponent includes the following six activity items: trips and visits, organised leisure, cleaning at home, health centre (visits to), gathering supplies and shopping. It involves events or pursuits that a group of people are doing together in a structured way. This implies that there is a collective structure to the activity rather than it being performed as an individual activity.

#### Subcomponent 2: Social activities and taking care of others

This subcomponent includes the following seven activity items: playing with others, caring for family, spiritual activities, celebrations, caring for animals or pets, social activities and meal preparation. It involves events or pursuits that bring members of the community together.

#### Subcomponent 3: Family life activities

This subcomponent includes the following three activity items: family time, family mealtime and quiet leisure. It involves events or pursuits that bring members of the family together.

#### Subcomponent 4: Personal care and development activities

This subcomponent includes the following three activity items: school, personal care and my own health. It refers to both basic self-care tasks of bathing, dressing, personal hygiene and grooming, as well as more complex tasks related to health and education.

### Internal consistency

The internal consistency calculated by Cronbach’s alpha for the total scale was 0.85. Cronbach’s alpha for the subcomponent *organised activities* was 0.72, for *social activities and taking care of others*, it was 0.75, for *family life activities*, it was 0.57 and for *personal care and development activities*, it was 0.49. This indicates that the internal consistency was adequate for the total scale and for the two subcomponents, *organised activities* and *social activities, and taking care of others* but questionable for the two subcomponents, *family life activities* and *personal care and development activities* (Terwee et al. 2007).

## Discussion

The main aim of this study was to explore the structural validity of the PMP instrument. The results of the PCA identified four subcomponents, namely, *organised activities, social activities and taking care of others, family life activities* and *personal care and development activities.* Following a discussion by an expert panel, these four subcomponents were confirmed as relevant ways of clustering activities into subcomponents of participation for children. Internal consistency was acceptable for the total scale and for two of the four subcomponents: *organised activities* and *social activities and taking care of others*. For the other two subcomponents – *family life activities* and *personal care and development activities* – internal consistency was lower. This is partly explained by the low number of items in those two scales, which makes them sensitive to small variations. Another explanation might be related to how latent constructs were defined. The labels of the subcomponents were partly based on whether the included items seemed to occur within an overarching context (such as activities in an everyday family context for the subcomponent family life activities) or with an assumed underlying purpose to strengthen the child’s autonomy in everyday life (such as in personal care and developmental activities). Hence, the labels strived to reflect a common theme of the included items, but did not necessarily indicate that they shared conceptually strong links. Taking care of personal hygiene is, for example, not strongly related to attending school, but children being independent in personal hygiene more frequently tend to attend school. The link to patterns of attendance rather than to only a psychometric ‘similarity’ illustrates the need for adopting a clinimetric approach as a supplementary aspect to psychometric properties (Fava, Tomba & Sonino 2012; Feinstein 1983; Marx et al. 1999). The aim of a psychometric approach is to develop scales that measure single perceived characteristics that have a resemblance in what they signify and such scales should be considered homogeneous (Hwang et al. 2013; Marx et al. 1999; Nunally & Bernstein 1994). The essence of a clinimetric approach is its reliance on the perceptions of informants, patients and clinicians concerning similarities using every day reasoning (Feinstein 1983; Marx et al. 1999) as discussed here.

In addition to the relatively high structural stability (high component loadings) that the items had in these subcomponents, they also made sense clinimetrically. These two subcomponents may consist of items that from a clinical perspective are interrelated (non-routine activities taking place in the home and personal routines), but they may not have strong relationships at an item level.

When evaluating scale properties using both statistical and theoretical perspectives, often, there are choices to be made that can influence which items will be clustered together into a scale. In this study, a four-component solution in the PCA was supported by both theoretical and statistical perspectives. Statistically, a two-component solution is probably as good as a four-component solution (see scree plot, Figure 1). However, if two components were used, they would have been so broad that they might be difficult to use for identifying and explaining patterns in participation such as differences in attendance between family life activities and organised leisure. According to the scree plot (Figure 1), the four-component solution is statistically adequate and it was clinically appropriate. In many participation measures, items are divided based on where the rated activities take place – at home or in society – and/or based on the type of activity, for example, leisure activity and social activities (Adair et al. 2018). This type of ‘pre-determined’ categorisation sometimes makes it difficult to create independent components with strong evidence for latent constructs. As can be seen in Table 4, some items had relatively higher statistical loadings (i.e. the item correlates relatively high with the subcomponent) on more than one component, for example, the item ‘mealtime preparation’ loads moderately both on subcomponent 2 (*social activities and taking care of others*) and on subcomponent 3 (*family life activities*). This forces instrument developers to think not only in terms of psychometric properties but also about the way in which items should be clustered in subcomponents to make clinical and practical sense. This clinimetric approach (Fava et al.2012; Feinstein 1983; Marx et al. 1999) may lead to some items being grouped in a subscale based on a certain purpose, for example, leisure activities, without those items having a necessarily high statistical relationship to the subscale. To illustrate, it is not probable that a high frequency of collecting stamps will be strongly related to a high frequency of playing football, although both can be categorised as leisure activities. It is argued that when questionnaires focused on measuring participation are developed, both the clinimetric and psychometric properties are considered together. The identified four subcomponents are discussed from the perspective of psychometric properties, latent constructs, clinical utility and clinimetric properties.

Firstly, the subcomponent *organised activities* contains six items with only one item having a component loading lower than 0.5, indicating that *organised activities* was a psychometrically sound subcomponent. All the items have one thing in common – they ask about organised activities that have a clear aim and a distinct beginning and end. Many of these activities are performed by a small group of people. The activities occur regularly but might not be scheduled. Children with mild ID can attend these activities and can be assigned tasks of different complexity, based on the skills and functioning level required by the activities. Thus, this subcomponent also seems to have clinimetric relevance because it may inform clinicians about what activities to target for intervention by discussing the result of the assessment with children and care providers.

Secondly, the subcomponent *social activities and taking care of others* contains seven items, of which five have a component loading of 0.5 or higher, which indicates relatively sound psychometric support for the subcomponent. What the items have in common is that they contain a social interactive aspect. However, one item (‘meal preparation’) also has a relatively high loading on the subcomponent *family life activities*. The item ‘mealtime’ also loads relatively high on family activities and ‘mealtime preparation’ can occur as a household task even if you are alone. However, as this measure is developed for children, it makes sense that children approach the preparing of food potentially in collaboration with others and as an opportunity to socialise.

Thirdly, the subcomponent *family life activities* contains three items. All of these items describe activities that occur in a family environment and probably involve close family. All items have a component loading of over 0.5 and illustrate a subcomponent that makes sense both psychometrically and clinimetrically. Both family activities (family time and family mealtime) containing social interactions with family members and an activity that probably occurs without much ongoing social interaction, ‘Quiet leisure’, are included. The activities are characterised by the child participating in activities in the home that are outside routines and organised activities.

The last subcomponent, *personal care and development activities,* contains three items all focused on taking care of your own person (health, personal care and school), with ‘My own health’ having a lower loading (0.4) than the other two items. Overall, the psychometric soundness is somewhat lower than for the other subcomponents. Clinimetrically, the subcomponent makes sense, although in a latent way. ‘School’ has a high loading together with ‘personal care’ and indicates an environment that is supportive of routines and autonomy probably enhances all individual routine activities.

The PMP was used to gather the child respondents’ own views about their participation in everyday activities. In developing the instrument, special focus was placed on making sure that children with cognitive problems, which might affect their understanding of items and scales in a questionnaire, could participate in the study. This was established by the three trial items in first step of the PMP procedure. This procedure facilitated the establishment of the children’s understanding of the concepts and of understanding the instructions. Another focus was to make sure that the items asked were relevant in low-resource settings and for varying cultural groups. For this reason, heterogeneous groups of children were sought for the validation: those with ID, TD, from different countries and from both high-income and low- or middle-income settings, across a fairly broad age group (7 to 18 years). However, another less explicit but shared characteristic was that all the participants, in different ways, experienced participation and participation restrictions in their everyday lives. The need and right to experience participation can be considered universal amongst all children and according to the WHO and United Nations Childrens Fund (UNICEF), all children also have the right to express their perceptions of such participation (Carroll-Lind et al. 2011; United Nations 2006; United Nations General Assembly 1989; WHO 2001, 2007). Data from this study, in which children with varying levels of ID across varying cultural settings were able to complete the PMP and thereby report on their own participation attendance, suggest that PMP can be a useful tool for understanding and targeting participation outcomes.

## Strengths and limitations

In designing the study, consideration was given to reducing the risk of bias, including sampling adequacy (sample size > 100 and seven times the number of items), appropriateness of the analysis methods and clarity of description of procedures (Mokkink et al. 2010a). The sampling strategy of this study was to strive for variation in terms of age, gender, country and/or context, socioeconomical circumstances, etc. This variation might have limited the psychometric outcomes. A larger sample within each ‘group’, rather than reducing the variation, would have been the best way to produce a more robust instrument in terms of psychometric properties. It can, however, also be a strength to test the validity of PMP in diverse contexts.

## Future directions

This study contributes evidence of validity in children with mild ID: future directions include testing validity in other disability groups. It is challenging to assess participation in children with disabilities (Coster & Khetani 2008; Lygnegard et al. 2013). To generate knowledge about children’s perceived participation, the assessment method has to be individually adapted, for example, to deal with special needs related to communication problems and to the child’s everyday life. Another challenge is to be ethically cautious about the validity of an instrument. Concerning the PMP, further research is needed, concerning adjustment for use with children with disabilities other than ID and recruitment of larger samples to allow for performing a confirmatory factor analysis to validate the identified subcomponents. In addition, further evidence of stability of the items and scales is needed. As stability may be affected by the natural variation of a child’s everyday functioning in different life situations related to environmental factors and the variation in children’s interests and preferences (AAIDD 2010), future test-retest reliability studies will need to consider how to control these factors. Usefulness of PMP for child-centred clinical purposes in intervention planning rather than as a screening tool also needs to be trialled.

## Conclusion

In this study, the structural validity of the PMP was explored by identifying possible subcomponents in the attendance scale and examining internal consistency of the total score and of each subcomponent. An exploratory PCA extracted four subcomponents: organised activities, social activities and taking care of others, family life activities and personal care and development activities. Internal consistency for the total scale and the first two subcomponents were acceptable. The four subcomponents of PMP can be used to provide information about possible domains in which participation and participation restrictions occur.
